# Reduction of CT artifacts from cardiac implantable electronic devices using a combination of virtual monoenergetic images and post-processing algorithms

**DOI:** 10.1007/s00330-021-07746-8

**Published:** 2021-02-25

**Authors:** Lenhard Pennig, David Zopfs, Roman Gertz, Johannes Bremm, Charlotte Zaeske, Nils Große Hokamp, Erkan Celik, Lukas Goertz, Marcel Langenbach, Thorsten Persigehl, Amit Gupta, Jan Borggrefe, Simon Lennartz, Kai Roman Laukamp

**Affiliations:** 1 Institute for Diagnostic and Interventional Radiology, Faculty of Medicine and University Hospital Cologne, University of Cologne, Cologne, Germany; 2 Department of Radiology, University Hospitals Cleveland Medical Center, Cleveland, OH USA; 3 Department of Radiology, Case Western Reserve University, Cleveland, OH USA; 4 Department of Radiology, Neuroradiology and Nuclear Medicine, Johannes Wesling University Hospital, Ruhr University Bochum, Bochum, Germany; 5 Department of Radiology, Massachusetts General Hospital, Harvard Medical School, Boston, MA USA; 6 Else Kröner Forschungskolleg Clonal Evolution in Cancer, University Hospital Cologne, Cologne, Germany

**Keywords:** Tomography, X-ray computed, Artifacts, Pacemaker

## Abstract

**Objectives:**

To evaluate the reduction of artifacts from cardiac implantable electronic devices (CIEDs) by virtual monoenergetic images (VMI), metal artifact reduction (MAR) algorithms, and their combination (VMI_MAR_) derived from spectral detector CT (SDCT) of the chest compared to conventional CT images (CI).

**Methods:**

In this retrospective study, we included 34 patients (mean age 74.6 ± 8.6 years), who underwent a SDCT of the chest and had a CIED in place. CI, MAR, VMI, and VMI_MAR_ (10 keV increment, range: 100–200 keV) were reconstructed. Mean and standard deviation of attenuation (HU) among hypo- and hyperdense artifacts adjacent to CIED generator and leads were determined using ROIs. Two radiologists qualitatively evaluated artifact reduction and diagnostic assessment of adjacent tissue.

**Results:**

Compared to CI, MAR and VMI_MAR_ ≥ 100 keV significantly increased attenuation in hypodense and significantly decreased attenuation in hyperdense artifacts at CIED generator and leads (*p* < 0.05). VMI ≥ 100 keV alone only significantly decreased hyperdense artifacts at the generator (*p* < 0.05). Qualitatively, VMI ≥ 100 keV, MAR, and VMI_MAR_ ≥ 100 keV provided significant reduction of hyper- and hypodense artifacts resulting from the generator and improved diagnostic assessment of surrounding structures (*p* < 0.05). Diagnostic assessment of structures adjoining to the leads was only improved by MAR and VMI_MAR_ 100 keV (*p* < 0.05), whereas keV values ≥ 140 with and without MAR significantly worsened diagnostic assessment (*p* < 0.05).

**Conclusions:**

The combination of VMI and MAR as well as MAR as a standalone approach provides effective reduction of artifacts from CIEDs. Still, higher keV values should be applied with caution due to a loss of soft tissue and vessel contrast along the leads.

**Key Points:**

*• The combination of VMI and MAR as well as MAR as a standalone approach enables effective reduction of artifacts from CIEDs.*

*• Higher keV values of both VMI and VMI*_*MAR*_ *at CIED leads should be applied with caution since diagnostic assessment can be hampered by a loss of soft tissue and vessel contrast.*

*• Recommended keV values for CIED generators are between 140 and 200 keV and for leads around 100 keV.*

**Supplementary Information:**

The online version contains supplementary material available at 10.1007/s00330-021-07746-8.

## Introduction

Cardiovascular implantable electronic devices (CIEDs) such as permanent pacemakers, implantable cardioverter defibrillators, and cardiac resynchronization therapy devices improve outcome of various cardiac diseases and are increasingly used in aging populations [1, 2]. Imaging of CIEDs is usually conducted after implantation and when complications—e.g., macrodislocation lead-dysfunctioning syndrome—are suspected. Conventional radiography represents the standard of care to evaluate physical integrity and positioning [2–4] with multidetector computed tomography (MDCT) of the chest being performed less frequently, e.g., for procedural planning of device lead extraction and assessment of lead perforation [5].

Metal artifacts arise as a combination of beam-hardening which results from absorption of low energetic photons [6, 7], photon starvation which is caused by an insufficient amount of photons reaching the detector [7, 8], and scatter artifacts [9]. In CT scans, the metallic generator and leads of implanted CIEDs may cause hypo- and hyperdense artifacts, which impede assessment of adjacent structures. For instance, artifacts surrounding the pectoral CIED generator can hamper assessment of surrounding vessels, soft tissue, and lymph nodes [2] with the latter being especially of importance in oncologic patients, in which detection of lymph node or muscle metastases is relevant [10]. Likewise, pacemaker leads can cause strong artifacts peaking at the lead tip, which impair the assessment of vessel lumen and image interpretation of cardiac structures, such as chambers, valves, myocardium, and major thoracic vessels [2]. Consequently, the detection of lead-associated thrombosis, coronary/valve calcification, myocardial hypertrophy, and pericardial effusion can thereby be hampered [11, 12].

Previous studies have demonstrated the use of virtual monoenergetic images (VMI) from spectral detector CT (SDCT) and metal artifact reduction algorithms (MAR) for reduction of artifacts from implanted metal material as standalone techniques and as combined approaches (VMI_MAR_) [12–17]. To date, in SDCT imaging the combination of both methods has not been evaluated to reduce artifacts arising from CIEDs.

The objective of the study was to investigate the potential of VMI, MAR, and VMI_MAR_ derived from venous phase SDCT of the chest to reduce artifacts surrounding CIED generator and leads. To this end, artifact reduction was objectively evaluated by examining the attenuation, or Hounsfield units (HU), respectively, of specific regions of interests (ROIs) around the CIED generator and leads. Additionally, artifact reduction was subjectively rated by two independent readers.

## Methods

This retrospective study was approved by the local institutional review board (reference number 20-1068) and conducted in accordance with the ethical regulations of the 1964 Helsinki declaration including later amendments. The local institutional review board waived the necessity for informed consent.

### Patient population

Patient scans were retrospectively selected from our internal database including data between March and December 2019 applying the following inclusion criteria:

(i) Contrast-enhanced, venous phase SDCT examinations of the thorax
(ii) Presence of a CIED with pectoral placement of the CIED generator and at least one lead with the respective tip positioned in a heart chamber
(iii) Availability of MAR reconstructions in addition to conventional and spectral image reconstructions
(iv) Age: ≥ 18 years

There were no explicit exclusion criteria.

### Imaging protocol

All patients were scanned head-first supine on a clinical SDCT (IQon, Philips Healthcare). No scans were explicitly performed for the purpose of this study. Iodinated contrast media (Accupaque 350 mg/ml, GE Healthcare) was administered into an antecubital vein with scans being initialized with a 20- (thorax) or 50-s delay (thorax and abdomen) after exceeding a threshold value of 150 HU in the descending aorta. The following scan parameters were used: matrix 512 × 512, collimation 64 × 0.625 mm, rotation time (thorax) 0.4 s / (thorax and abdomen) 0.33 s, pitch (thorax) 1.015 / (thorax and abdomen) 0.671, tube voltage 120 kVp. All examinations were conducted using automatic tube current modulation (DoseRight 3D-DOM, Philips Healthcare). Dose right index for the thorax examinations was 13 and for the thoracoabdominal examinations 17.

### Image post-processing

Image reconstructions were performed using a hybrid-iterative reconstruction algorithm with a standard soft tissue kernel using the following specifications:

1. Conventional CT images (iDose4, level 3, filter B, Philips Healthcare; referred to as CI)
2. MAR (O-MAR, filter B, Philips Healthcare)
3. VMI (spectral B, level 3, Philips Healthcare; range: 100–200 kiloelectron volt (keV), increment of 10 keV)
4. Combination of MAR and VMI (spectral B, level 3, range: 100–200 keV, increment of 10 keV; referred to as VMI_MAR_).

For all reconstructions, slice thickness was set to 2 mm with an overlap of 50%. MAR reconstructions were only performed on demand when artifacts from CIED were impairing diagnostic assessment. On the contrary, VMI reconstructions can be derived from SDCT after every examination.

### Objective analysis

Image assessment was performed by a radiologist with 3 years of experience in chest imaging using a ROI-based method with a consistent size of approximately 50 mm^2^. ROIs were placed in CI and copied to MAR, VMI, and VMI_MAR_ using the vendor’s proprietary image viewer (IntelliSpace Portal, v9; Philips Healthcare).

ROIs were located in pronounced hypo- and hyperdense artifacts surrounding the CIED generator and the tip of CIED leads. Furthermore, for each ROI in artifact-impaired tissue, a ROI in the corresponding artifact-free reference tissue was also placed. For instance, when a hyperdense artifact impaired the ventricle lumen, an artifact-free region within the ventricle lumen was selected as the reference tissue. For each patient, seven ROIs were placed each in CI and MAR (reference tissue for artifact-free ventricle lumen could be used for the hypo- and hyperdense artifact around the tip of CIED leads) and then copied to the VMI at the reconstructed keV levels. The mean and standard deviation (SD) of attenuation within each ROI was recorded. We considered the SD in artifact-impaired tissue indicative for artifact burden [18], although it needs to be considered that SD depends on several factors that might bias artifact reduction and although changes in SD can be rather subtle even though changes in mean attenuation can be large.

The corrected attenuation for hypo- and hyperdense artifacts was calculated as the difference of attenuation in artifact-impaired and artifact-free reference tissue [13]. This method considers general changes in attenuation along altering keV values of VMI to minimize potential bias to only detect real artifacts and artifact reduction [19]. Furthermore, corrected image noise [20] was calculated as the difference between image noise in artifact-impaired and artifact-free reference tissue. This method accounts for the general lower image noise in VMI with higher keV [19].

### Subjective analysis

Two radiologists with 2 and 3 years of experience in chest imaging independently evaluated images on the same Picture Archiving and Communication System workstation (IMPAX EE release 20; Agfa HealthCare N.V.). Readers were blinded to clinical and patient data as well as to the results of the objective analysis. Full blinding towards reconstructions appeared not feasible for a consistent evaluation because of their distinct visual characteristics. Therefore, in each patient, readers were given a complete set of images, including CI, VMI, and VMI_MAR_. KeV values for VMI and VMI_MAR_ were 100, 140, and 200 keV. The larger increments compared to the objective analysis were chosen to allow for detection of relevant changes in image assessment. These might otherwise be obscured when images are rated that are too similar due to smaller keV value increment. Image parameters were as follows: axial plane, initial window level: 60, and window width: 360. Readers were allowed to adjust the window settings manually.

Readers were instructed to assess the extent of hypo- and hyperdense artifacts surrounding CIED generator and leads using a 5-point Likert scale (Table 1). Alike, they evaluated the diagnostic assessment of tissue adjacent CIED generator as well as cardiac structures and major vessels surrounding the CIED leads on a 5-point Likert scale (Table 1); readers considered artifact reduction capabilities but also a loss of soft tissue/vessel contrast that can appear at higher keV values in VMI and new artifacts that can be introduced by application of VMI as well as MAR [13, 21, 22].

**Table 1:** Likert scale for subjective analysis of artifact reduction. *CIED*, cardiac implantable electric device; *CI*, conventional images; *MAR*, metal artifact reduction algorithm

Extent of hypo- and hyperdense artifacts surrounding CIED generator and leads	(5) Artifacts are absent/almost absent
	(4) Minor artifacts
	(3) Moderate artifacts
	(2) Pronounced artifacts
	(1) Massive artifacts
Diagnostic assessment of pectoral soft tissue surrounding CIED generator, e.g., lymph nodes, muscles, fat, and vessels	(5) Fully diagnostic quality by no artifacts/almost no artifacts
	(4) Marginally affected diagnostic interpretability by minor streaks
	(3) Hampered diagnostic interpretability by moderate artifacts
	(2) Restricted diagnostic interpretability by strong artifacts
	(1) Insufficient diagnostic interpretability
Diagnostic assessment of the heart and major associated vessels adjacent to CIED leads regarding heart chambers, myocardium, and pericardium, also considering potential cardiac pathologies, e.g., thrombosis, calcification, myocardial hypertrophy, and pericardial effusion	(5) Full diagnostic quality/certainty without artifacts/almost no artifacts
	(4) Marginally affected diagnostic quality/certainty by minor streaks
	(3) Hampered diagnostic quality/certainty by moderate artifacts
	(2) Restricted diagnostic quality/certainty by strong artifacts
	(1) Insufficient diagnostic quality/certainty

### Statistical analysis

Statistical analysis was performed with JMP software (release 14.1.0, SAS Institute). Quantitative results are shown as mean ± SD with qualitative results being displayed as median with 10/90 percentile. The Shapiro-Wilk test was used to test for normal distribution. The Wilcoxon test with Steel adjustment for multiple comparisons was performed to test for significant differences. The statistical significance value was defined as *p* < 0.05. Interreader agreement was assessed by calculating the intraclass correlation coefficient (ICC). Agreement was considered excellent when ICC > 0.74, good when ICC = 0.60–0.74, fair when ICC = 0.40–0.59, and poor when ICC < 0.4 [23].

## Results

### Study population and baseline characteristics

Thirty-four patients were included in this study (mean age 74.6 ± 8.6 years, 11 females). Seven patients had a single-chamber implanted cardioverter defibrillator (ICD), 26 patients a dual-chamber ICD, and one patient had a cardiac resynchronization therapy device placed. The manufacturers and models of the CIED generator and leads are provided in Table 1 of the supplementary material. CT scans of the thorax were performed in one patient, and of the abdomen and thorax in 33 patients.

### Objective analysis

#### CIED generator

**Fig. 1:**
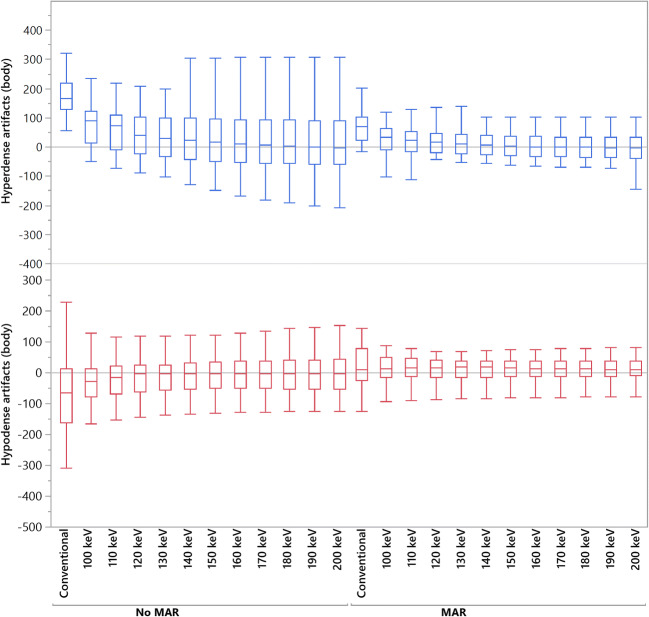
Box-plot diagram displaying corrected attenuation values within hypo- and hyperdense artifacts adjacent to CIED generator in conventional CT images (conventional), virtual monoenergetic images (VMI, 100–200 keV), metal artifact reduction (MAR) algorithms, and their combination. HU, Hounsfield units

Results of objective analysis of artifact reduction at CIED generator are provided in Table 2 and Fig. 1. Compared to CI, in higher keV of VMI, the corrected attenuation within hypodense artifacts was increased (e.g., CI/VMI_200keV_: − 78.9 ± 129.5/4.3 ± 119.6 HU, *p* > 0.05) and decreased within hyperdense artifacts (e.g., CI/VMI_200keV_: 171.4 ± 165.4/32.3 ± 189.8 HU, *p* < 0.05), for the latter with statistical significant differences at ≥ 100 keV. MAR and VMI_MAR_ ≥ 100 keV significantly increased/decreased corrected attenuation in hypo-/hyperdense artifacts (*p* < 0.05).

**Table 2:** Objective analysis of artifact reduction and surrounding tissues at CIED generator. Data is reported as mean ± SD. *CI*, conventional images; *VMI*, virtual monoenergetic images; *MAR*, metal artifact reduction algorithm; *VMI*_*MAR*_, combination of MAR and VMI. Bold indicates significant changes in HU values compared to CI

	Corrected attenuation		Corrected image noise	
	Hypodense artifacts	Hyperdense artifacts	Hypodense artifacts	Hyperdense artifacts
CI	− 78.9 ± 129.5	171.4 ± 165.4	69.9 ± 54.9	55.2 ± 92.0
VMI				
100 keV	− 37.9 ± 97.8	**96.6 ± 145.6**	56.9 ± 62.2	50.7 ± 57.8
140 keV	− 8.8 ± 110.2	**52.3 ± 173.5**	50.6 ± 64.9	46.4 ± 53.8
200 keV	4.3 ± 119.6	**32.3 ± 189.8**	49.4 ± 66.3	46.3 ± 52.5
MAR	**18.4 ± 69.5**	**70.1 ± 50.0**	**13.3 ± 13.2**	**17.0 ± 13.2**
VMI_MAR_				
100 keV	**10.2 ± 46.2**	**28.3 ± 50.0**	**8.5 ± 9.0**	**13.0 ± 11.2**
140 keV	**10.1 ± 40.7**	**10.5 ± 56.2**	**6.5 ± 8.4**	**10.8 ± 10.8**
200 keV	**10.0 ± 39.8**	**2.5 ± 59.7**	**5.8 ± 8.3**	**10.1 ± 10.7**
*p* value				
CI vs. VMI 100–200 keV	> 0.05	< 0.05	> 0.05	> 0.05
CI vs. MAR	< 0.05	< 0.05	< 0.05	< 0.05
CI vs. VMI_MAR_ 100–200 keV	< 0.05	< 0.05	< 0.05	< 0.05

VMI provided decreased corrected image noise in hypo- and hyperdense artifacts at all keV values, albeit this effect was not statistically significant (*p* > 0.05). However, MAR (*p* < 0.05) and VMI_MAR_ ≥ 100 keV (*p* < 0.05) enabled a significant reduction of corrected image noise in hypo- and hyperdense artifacts.

#### CIED leads

**Fig. 2:**
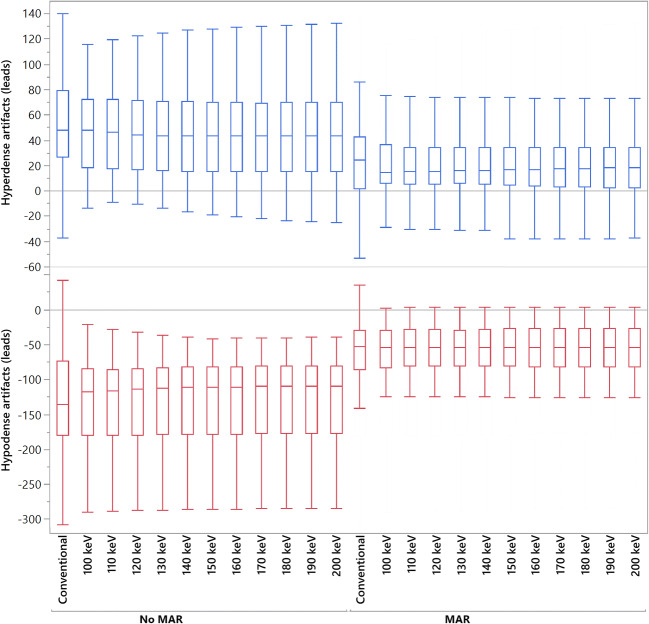
Box-plot diagram displaying corrected attenuation values within hypo- and hyperdense artifacts adjacent to CIED leads in conventional CT images (conventional), virtual monoenergetic images (VMI, 100–200 keV), metal artifact reduction (MAR) algorithms, and their combination. HU, Hounsfield units

Results of objective analysis of artifact reduction around CIED leads are given in Table 3 and Fig. 2. Compared to CI in VMI ≥ 100 keV, corrected attenuation in hypo- and hyperdense artifacts was comparable without yielding statistical significance (*p* > 0.05). MAR alone provided a significant increase of attenuation in hypodense (CI/MAR: − 127.5 ± 77.3/− 59.7 ± 50.4 HU; *p* < 0.05) and reduction in hyperdense (CI/MAR: 51.8 ± 37.7/22.3 ± 30.5; *p* < 0.05) artifacts. Likewise, VMI_MAR_ ≥ 100 keV yielded a significant increase and decrease in corrected attenuation for hypo- and hyperdense artifacts, respectively (*p* < 0.05).

**Table 3:** Objective analysis of artifact reduction and surrounding tissues at CIED leads. Data is reported as mean ± SD. *CI*, conventional images; *VMI*, virtual monoenergetic images; *MAR*, metal artifact reduction algorithm; *VMI*_*MAR*_, combination of MAR and VMI. Bold indicates significant changes in HU values compared to CI

	Corrected attenuation		Corrected image noise	
	Hypodense artifacts	Hyperdense artifacts	Hypodense artifacts	Hyperdense artifacts
CI	− 127.5 ± 77.3	51.8 ± 37.7	66.5 ± 46.1	29.3 ± 24.3
VMI				
100 keV	− 128.2 ± 64.9	47.6 ± 32.9	65.4 ± 41.7	30.1 ± 24.4
140 keV	− 127.2 ± 62.6	47.0 ± 35.5	64.9 ± 41.8	29.3 ± 24.8
200 keV	− 126.8 ± 61.9	46.7 ± 37.0	64.7 ± 42.1	29.1 ± 25.1
MAR	**− 59.7 ± 50.4**	**22.3 ± 30.5**	**23.6 ± 23.2**	**9.3 ± 12.9**
VMI_MAR_				
100 keV	− **58.2 ± 39.4**	**19.7 ± 24.6**	**21.2 ± 21.4**	**10.6 ± 10.7**
140 keV	− **57.1 ± 38.0**	**18.8 ± 24.3**	**20.4 ± 22.1**	**10.0 ± 10.1**
200 keV	− **56.5 ± 37.8**	**18.4 ± 24.7**	**20.1 ± 22.4**	**9.7 ± 9.9**
*p* value				
CI vs. VMI 100–200 keV	> 0.05	> 0.05	> 0.05	> 0.05
CI vs. MAR	< 0.05	< 0.05	< 0.05	< 0.05
CI vs. VMI_MAR_ 100–200 keV	< 0.05	< 0.05	< 0.05	< 0.05

Corrected image noise in CI and VMI ≥ 100 keV was comparable in hypo- and hyperdense artifacts (*p* < 0.05). However, MAR and VMI_MAR_ ≥ 100 keV provided a significant decrease of image noise in both hypo- and hyperdense artifacts.

### Subjective analysis

#### CIED generator

Results of subjective analysis of artifact reduction and surrounding tissue at CIED generator are given in Table 4. VMI ≥ 100 keV, MAR, and VMI_MAR_ provided significant reduction of hypo- and hyperdense artifacts compared to CI (*p* < 0.05). Diagnostic assessment of tissue adjacent to CIED generator significantly improved in VMI ≥ 100 keV, MAR, and VMI_MAR_ at 140 keV and 200 keV.

**Table 4:** Subjective analysis of artifact reduction and presence of new artifacts at CIED generator. Data is reported as median with 10/90 percentile. *CI*, conventional images; *VMI*, virtual monoenergetic images; *MAR*, metal artifact reduction algorithm; *VMI*_*MAR*_, combination of MAR and VMI; *ICC*, intraclass correlation coefficient. Bold indicates significant changes in scores compared to CI

	Artifact extent		Diagnostic assessment of surrounding tissue
	Hypodense	Hyperdense	
CI	3 (1–3)	3 (1–4)	3 (1–3)
VMI			
100 keV	**3 (2–4)**	**3 (2–5)**	**3.5 (2–4)**
140 keV	**4 (2–5)**	**4 (2–5)**	**4 (2–5)**
200 keV	**4 (2–5)**	**4 (2–5)**	**4 (2–5)**
MAR	**3 (2–4)**	**4 (3–4)**	**3 (3–4)**
VMI_MAR_			
100 keV	**4 (3–5)**	**4 (3–5)**	**4 (3–5)**
140 keV	**5 (3–5)**	**5 (4–5)**	**5 (4–5)**
200 keV	**5 (3–5)**	**5 (4–5)**	**5 (4–5)**
*p* value			
CI vs. VMI 100 keV	< 0.05	< 0.05	< 0.05
CI vs. VMI 140 keV	< 0.05	< 0.05	< 0.05
CI vs. VMI 200 keV	< 0.05	< 0.05	< 0.05
CI vs. MAR	< 0.05	< 0.05	< 0.05
CI vs. VMI_MAR_ 100–200 keV	< 0.05	< 0.05	< 0.05

#### CIED leads

Table 5 displays the results of subjective analysis of artifact reduction and surrounding tissue at CIED leads. Only MAR and VMI_MAR_ ≥ 100 keV provided significant reduction of hypodense artifacts (*p* < 0.05), whereas for hyperdense artifacts, all three techniques (VMI ≥ 100 keV, MAR, and VMI_MAR_) enabled significant decrease of artifacts (*p* < 0.05). Only MAR, VMI at 100 keV, and VMI_MAR_ at 100 keV yielded a significant improvement for diagnostic assessment of tissue and cardiac structures adjacent to the leads. KeV values of 140 or higher in VMI and VMI_MAR_ led to worsened diagnostic assessment.

**Table 5:** Subjective analysis of artifact reduction and presence of new artifacts at CIED leads. Data is reported as median with 10/90 percentile. *CI*, conventional images; *VMI*, virtual monoenergetic images; *MAR*, metal artifact reduction algorithm; *VMI*_*MAR*_, combination of MAR and VMI; *ICC*, intraclass correlation coefficient. Bold indicates significant changes in scores compared to CI

	Artifact extent		Diagnostic assessment of surrounding tissue
	Hypodense	Hyperdense	
CI	3 (3–4)	2 (2–3)	2 (2–3)
VMI			
100 keV	3 (3–4)	**3 (2**–**4)**	**3 (2**–**3)**
140 keV	3 (3–4)	**3 (2**–**4)**	**2 (1**–**4)**
200 keV	3 (3–4)	**3 (3**–**4)**	**1 (1**–**4)**
MAR	**4 (3**–**5)**	**3 (2**–**4)**	**3 (2**–**4)**
VMI_MAR_			
100 keV	**4 (3**–**5)**	**4 (3**–**4)**	**3 (2**–**4)**
140 keV	**4 (3**–**5)**	**4 (3**–**5)**	**2 (1**–**4)**
200 keV	**4 (3**–**5)**	**4 (3**–**5)**	**1 (1**–**4)**
*p* value			
CI vs. VMI 100 keV	> 0.05	< 0.05	< 0.05
CI vs. VMI 140 keV	> 0.05	< 0.05	< 0.05
CI vs. VMI 200 keV	> 0.05	< 0.05	< 0.05
CI vs. MAR	< 0.05	< 0.05	< 0.05
CI vs. VMI_MAR_ 100–200 keV	< 0.05	< 0.05	< 0.05

Interreader agreement was good (ICC = 0.66).

**Fig. 3:**
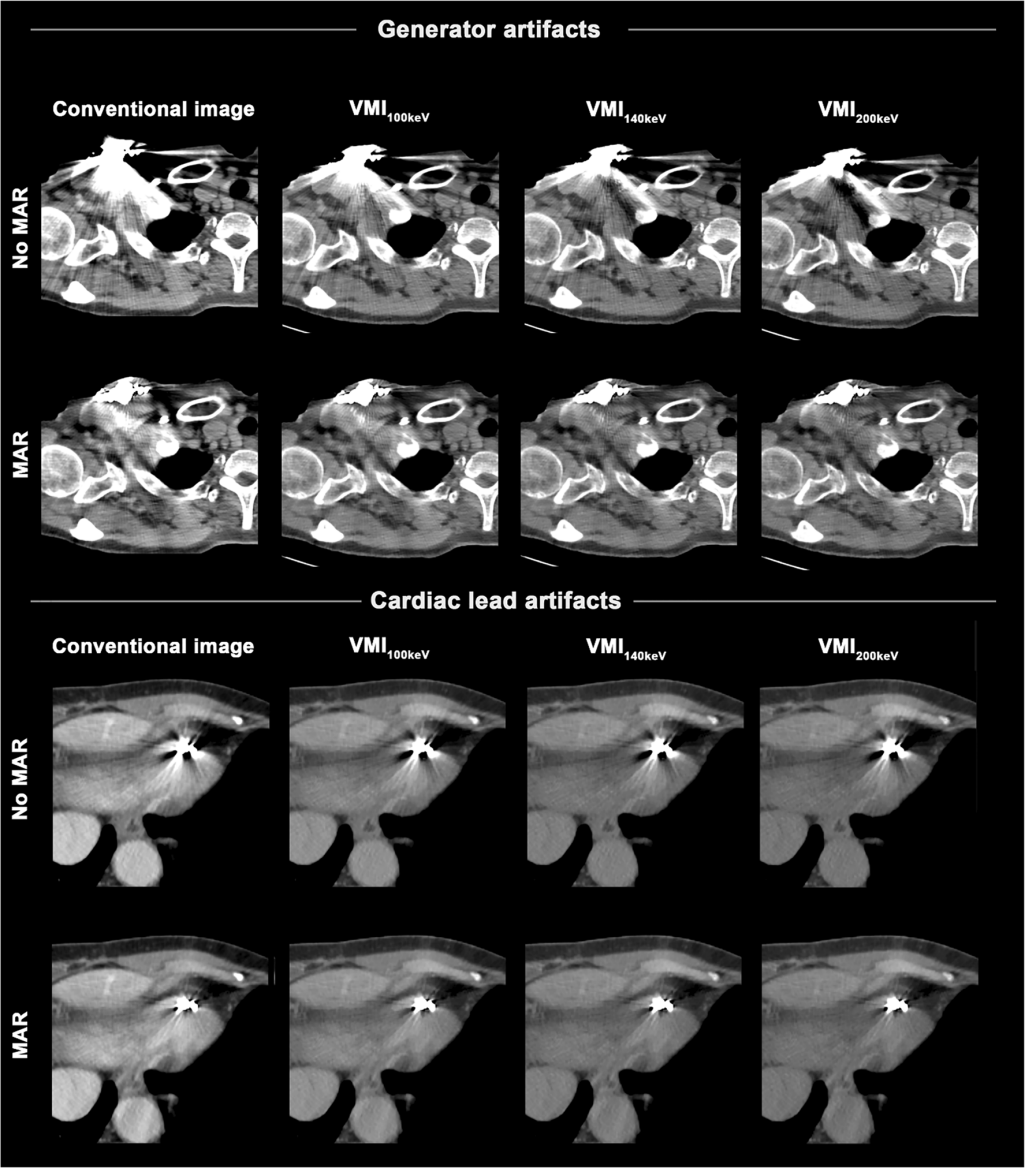
Conventional images, virtual monoenergetic images (VMI, 100–200 keV), MAR, and their combination (VMI_MAR_) in a 65-year-old female patient with a dual-chamber implanted cardioverter defibrillator in place. For the CIED generator, VMI as a standalone (top row) approach allow for reduction of hyperdense and hypodense artifacts with best performance at higher keV values, although relevant residual artifacts remain. These can be further reduced by MAR and VMI_MAR_ (second row). For the CIED leads, VMI alone (third row) provide only minimal benefit in artifact reduction, whereas performance by VMI_MAR_ (bottom row) is a lot stronger

An illustrative case of artifact reduction around CIED generator and leads is presented in Fig. 3.

## Discussion

In this study, we assessed the performance of VMI, MAR, and their combination (VMI_MAR_) for reduction of artifacts from CIED generator and leads in SDCT imaging.

As major findings of this study, MAR and VMI_MAR_ provided the most efficient artifact reduction in subjective and objective analysis while yielding improved diagnostic assessment of soft tissue and cardiac structures adjacent to CIED generator and leads. VMI at higher keV levels provided significant reduction of artifacts at CIED generator and leads (with exception of VMI for hypodense artifacts at the leads) in the subjective assessment, whereas a significant decrease of artifacts in the objective analysis was only observed for hyperdense artifacts at CIED generator. Of note, VMI and VMI_MAR_ led to relevant and significant decrease of diagnostic assessment at ≥ 140 keV at CIED leads.

Prior studies have investigated the use of VMI, MAR, and their combination (VMI_MAR_) from dual-energy CT for reduction of artifacts arising from high density materials, such as orthopedic hardware [24], dental implants [13], deep brain stimulation leads [25], iodinated contrast agent [26], and coils and clips for intracranial aneurysm treatment [21, 27, 28]. More recent studies focusing on the reduction of artifacts impairing the assessment of the heart and surrounding structures (e.g., from pacemaker devices or other cardiac hardware) showed promising results for the application of artifact reduction algorithms and reconstruction techniques to improve image quality [12, 14–17]. For instance, Tatsugami et al. demonstrated that artifact reduction algorithms allow for a superior assessment of coronary arteries in patients with CIEDs [12]. Also, the application of a convolutional network has been successfully tested for the reduction of artifacts from pacemaker leads [16]. Van Hedent et al. investigated the application of VMI for the reduction of artifacts in chest and abdominal imaging of patients including artifacts from pacemakers [17].

Considering these previous studies, there has been uncertainty whether either method on its own (VMI or MAR) can provide sufficient artifact reduction. Particularly for stronger artifacts, e.g., those arising from CIED generator and leads, MAR and especially VMI as standalone approaches might yield suboptimal artifact reduction [13, 25, 29–31]. In line with these findings, our study demonstrated that, although VMI reduced artifacts in the visual assessment, it failed to significantly reduce artifacts in quantitative measurements, except for hyperdense artifacts at CIED generator. In contrast, MAR and VMI_MAR_ enabled good artifact reduction for both kinds of artifacts and compartments of CIED as outlined in subjective and objective results. Still, this positive effect did not reflect on the diagnostic assessment of cardiac structures surrounding the CIED leads at higher keV values.

In line with previous studies, subjective artifact reduction at CIED generator and leads improved at higher keV levels in VMI and VMI_MAR_ [13, 32]. However, this effect does not necessarily result in improved diagnostic assessment. This is due to the physical properties of VMI that accompanies higher keV values by the greater distance to the k-edge of iodine (~33 keV) [19], which at higher keV levels reduce contrast of soft tissue, vessel lumen, and cardiac chambers [19, 33]. This loss of contrast was found less severe around the CIED generator; therefore, diagnostic assessment was best at high keV values of ≥140 keV. Hence, we recommend keV values between 140 and 200 keV to assess structures around the CIED generator. On the contrary, the loss of contrast of vessels and heart chambers significantly hampers the diagnostic assessment at higher keV values at the leads. Therefore, we recommend 100 keV for optimal diagnostic assessment of structures adjacent to CIED leads. Nevertheless, as the optimal keV value differs between patients, these settings may need to be adjusted individually. A known limitation of VMI and MAR algorithms is that they can cause/produce new artifacts, which can impact the diagnostic quality of CT images [13, 21, 22]. Hence, we recommend the use of VMI and MAR only when artifacts are present, and CI should remain the standard of care. Of note, MAR was initially intended to be applied for orthopedic hardware. Still, as eluded above, several studies have shown its effectiveness also for non-orthopedic hardware [13, 21, 25, 34], e.g., deep brain stimulating electrodes [25]. However, in its white paper [22], the vendor does not recommend to apply MAR when metal is near air or low density tissue, e.g., in pacemakers, as the proximity to the lung can induce new artifacts [22]. Also, in smaller metal objects such as cardiac stents, the MAR algorithm might not alter image information as no or minimal metal is present [22, 25]. To this point, the vendor even disables MAR in dedicated cardiac examinations. Still, Große Hokamp et al. showed the dedicated value of MAR for reduction of artifacts from deep brain stimulating electrodes [25].

With the varying degrees of effectiveness for artifact reduction depending on implant type and its location within the human body, VMI and MAR algorithms for CT imaging are available from all major vendors [19, 35]. However, their combined use is only possible in dual-energy CT. Nevertheless, single-energy CT scanners are by far much more common than DECT in clinical routine; thereby, MAR as a standalone post-processing approach has the advantage of a higher availability in conventional CT systems [24, 35]. Given the results of this study, MAR, not only in combination with VMI but also standalone, enables good reduction of artifacts arising from CIED generator and leads, which allows usage on conventional non-dual-energy CT systems.

### Limitations

Besides its retrospective, single-center setting, our study has the following additional limitations that should be considered. First, the method to measure artifacts needs to be discussed. Due to its high feasibility, a relatively simple and standard approach in artifact reduction studies is ROI-based measurement of mean and standard deviation of attenuation [13, 36, 37]. Nevertheless, more elaborate methods using dedicated artifact quantification algorithms might produce more precise results [25, 38]. Second, given their differing physical properties, general changes in terms of attenuation and image noise appear in different keV levels of VMI [19]. To this end, we applied an intra-individual comparison between artifact-impaired tissue and correspondent unimpaired reference tissue resulting in corrected attenuation and corrected image noise, which enables the detection of real artifact reduction [13]. Third, full blinding of readers did not seem feasible since images (CI, VMI, and VMI_MAR_) are distinguishable by their appearance. Furthermore, we aimed to encourage readers to also detect more subtle differences between reconstructions next to a qualitative rating of images; therefore, a full image set of one patient at a time was presented to the readers.

## Conclusions

In presence of artifacts from CIED, the combination of VMI and MAR provides the most effective artifact reduction and improves diagnostic assessment of surrounding structures; however, MAR as a standalone approach also provides good artifact reduction. Still, higher keV values at CIED leads should be applied with caution due to a loss of soft tissue and vessel contrast, which impedes diagnostic accuracy. Based on our data, we recommend keV values between 140 and 200 keV for artifacts surrounding the CIED generator and of 100 keV for artifacts adjacent to the leads.

## Supplementary information

ESM 1 (DOCX 19 kb)

## Abbreviations

CI: Conventional CT images
CIED: Cardiovascular implantable electronic devices
ICC: Intraclass correlation coefficient
ICD: Implanted cardioverter defibrillator
keV: Kiloelectron volt
MAR: Metal artifact reduction
MDCT: Multidetector computed tomography
ROI: Region of interest
SD: Standard deviation
SDCT: Spectral detector CT
VMI: Virtual monoenergetic images
VMI_MAR_: Combination of MAR and VMI

## Declarations

### Guarantor

The scientific guarantors of this publication are Lenhard Pennig and Kai Roman Laukamp.

### Statistics and biometry

No complex statistical methods were necessary for this paper.

### Methodology

• retrospective

• observational

• performed at one institution
