# Supplementary material for: Reduction of CT artifacts from cardiac implantable electronic devices using a combination of virtual monoenergetic images and post-processing algorithms
Source: Eur Radiol. 2021 Feb 25;31(9):7151–61. doi: 10.1007/s00330-021-07746-8 (PMC8379133; doi:10.1007/s00330-021-07746-8)
Supplement: Supplementary file 1 — (DOCX 19 kb) [file 330_2021_7746_MOESM1_ESM.docx]

| System | Generator | RA | RV | Coronary sinus |
| --- | --- | --- | --- | --- |
| Dual-chamber ICD (RA, RV) | Boston Scientific, Essentio DRL101 | Medtronic, CapSureFix 5076-52cm | Medtronic, CapSureFix 5076-58cm | - |
| Dual-chamber ICD (RA, RV) | Medtronic, Sensia SEDR01 | Medtronic, CapSureFix 4076-52cm | Medtronic, CapSureSense 4074-58cm | - |
| Dual-chamber ICD (RA, RV) | Biotronik, Enticos 4 DR MR | Biotronik, Solia S 53 | Biotronik, Solia S 60 | - |
| Single-chamber ICD (RV) | Boston Scientific, Inogen | - | Boston Scientific, Endotak reliance 0292 | - |
| Dual-chamber ICD (RA, RV) | Medtronic, Sensia SEDR01 | Boston Scientific, Flextend 2 | Medtronic, CapSure SP 4092 | - |
| Dual-chamber ICD (RA, RV) | Medtronic, Ensura DR | Medtronic, CapSureFix 5076-52cm | Medtronic, CapSureFix 5076-58cm | - |
| Dual-chamber ICD (RA, RV) | n/a | n/a | n/a | - |
| Dual-chamber ICD (RA, RV) | Medtronic, Ensura EN1DR01 | Medtronic, CapSureFix 5076-52cm | Medtronic, CapSureFix 5076-58cm | - |
| Dual-chamber ICD (RA, RV) | Medtronic, Ensura DR | Medtronic, CapSureFix 4076-52cm | Medtronic, CapSureFix 4076-58cm | - |
| Dual-chamber ICD (RA, RV) | n/a | n/a | n/a | - |
| Dual-chamber ICD (RA, RV) | Biotronik, Ecuro DR | Biotronik, Solia S 53 | Biotronik, Solia S 60 | - |
| Dual-chamber ICD (RA, RV) | n/a | n/a | n/a | - |
| Dual-chamber ICD (RA, RV) | Biotronik, Etrinsa 8 DR-T | Biotronik, Solia S 53 | Biotronik, Solia S 60 | - |
| Dual-chamber ICD (RA, RV) | Medtronic, Sensia SEDR01 | Medtronic, CapSureFix 5076 | Medtronic, CapSureFix 5076 | - |
| Dual-chamber ICD (RA, RV) | St. Jude, Ellipse DR 2377-36QC | St. Jude, 2088TC Tendril STS | St. Jude, Durata 7122Q | - |
| Dual-chamber ICD (RA, RV) | Medtronic, Ensura EN1DR01 | Medtronic, CapSureFix 5076-52cm | Medtronic, CapSureFix 5076-58cm | - |
| Dual-chamber ICD (RA, RV) | Boston Scientific, Advantio J063 DR | Medtronic, CapSureFix 5076-52cm | Medtronic, CapSureFix 5076-58cm | - |
| Single-chamber ICD (RV) | Boston Scientific, Inogen | - | Boston Scientific, Endotak reliance 0292 | - |
| Dual-chamber ICD (RA, RV) | St. Jude, Accent MRI 2124 | n/a | n/a | - |
| Dual-chamber ICD (RA, RV) | n/a | n/a | n/a | - |
| Single-chamber ICD (RV) | Medtronic, Protecta VR D364VRG | - | Medtronic, Durata 7122 | - |
| Single-chamber ICD (RV) | Vitatron, Q20 A2 SR | - | Medtronic, CapSureFix 5076-58cm | - |
| Dual-chamber ICD (RA, RV) | n/a | n/a | n/a | - |
| Single-chamber ICD (RV) | Boston Scientific, Essentio SR | - | Medtronic, CapSureFix 5076-58cm | - |
| Dual-chamber ICD (RA, RV) | n/a | n/a | n/a | - |
| Dual-chamber ICD (RA, RV) | Boston Scientific, Essentio SR | Medtronic, CapSureFix 5076-52cm | Medtronic, CapSureFix 5076-58cm | - |
| Dual-chamber ICD (RA, RV) | Boston Scientific, Advantio | n/a | n/a | - |
| Dual-chamber ICD (RA, RV) | Boston Scientific, Essentio DRL101 | Medtronic, CapSureFix 5076-52cm | Medtronic, CapSureFix 5076-58cm | - |
| Single-chamber ICD (RV) | St. Jude, Ellipse VR | - | St. Jude, Durata 7122Q-65cm | - |
| Dual-chamber ICD (RA, RV) | Medtronic, Sensia SEDR01 | Medtronic, CapSureFix 5076-52cm | Medtronic, CapSureSense 4074-58cm | - |
| CRT-P (RA, RV, coronary sinus) | Boston Scientific, Contak Renewal TR2 | Boston Scientific, Flextend 2 4097 | Boston Scientific, Flextend 2 4097 | Boston Scientific, Easytrak 3 4525 |
| Single-chamber ICD (RV) | Boston Scientific, Essentio SR | - | Medtronic, CapSureFix 5076-58cm | - |
| Dual-chamber ICD (RA, RV) | n/a | n/a | n/a | - |
| Dual-chamber ICD (RA, RV) | n/a | n/a | n/a | - |

Supplementary Table 1: Manufacturers and models of the generators and leads included in this study. RA=right atrium. RV=right ventricle. ICD= implanted cardioverter defibrillator. CRT-P=cardiac resynchronization therapy pacemaker. n/a=not available.
